# Early Postoperative Hyperglycemia After Arthroplasty in Type 2 Diabetes: Insights from Continuous Glucose Monitoring and Identification of Predictive Glycemic Parameters

**DOI:** 10.3390/life15101594

**Published:** 2025-10-13

**Authors:** Toshiyuki Tateiwa, Jumpei Shikuma, Yasuhito Takahashi, Itaru Nakamura, Hajime Matsumura, Ryo Suzuki, Kengo Yamamoto

**Affiliations:** 1 Department of Orthopedic Surgery, Tokyo Medical University, Tokyo 160-0023, Japan; 2 Department of Diabetes, Metabolism and Endocrinology, Tokyo Medical University, Tokyo 160-0023, Japan; 3 Department of Bone and Joint Biomaterial Research, Tokyo Medical University, Tokyo 160-0023, Japan; 4 Department of Infection Prevention and Control, Tokyo Medical University Hospital, Tokyo 160-0023, Japan; 5 Department of Plastic and Reconstructive Surgery, Tokyo Medical University, Tokyo 160-0023, Japan

**Keywords:** lower extremity arthroplasty, type 2 diabetes mellitus, postoperative glycemic control, continuous glucose monitoring, glycemic variability

## Abstract

Background: Diabetes mellitus is a well-established risk factor for surgical site infections (SSIs), particularly periprosthetic joint infections (PJIs) following joint arthroplasty. Although strict glycemic control in the early postoperative period is critical, few studies have evaluated glycemic dynamics using continuous glucose monitoring (CGM) in this setting. This study aimed to characterize early postoperative glycemic patterns using CGM in patients with type 2 diabetes mellitus undergoing lower extremity arthroplasty and to identify factors associated with postoperative hyperglycemia. Methods: We retrospectively analyzed 41 patients with type 2 diabetes who underwent total hip or knee arthroplasty. CGM was used to monitor glucose levels continuously for 48 h after surgery. All patients received standard glycemic management based on a sliding-scale insulin protocol. Patients were classified into two groups: normoglycemia (glucose consistently < 200 mg/dL) and hyperglycemia (glucose ≥ 200 mg/dL at least once within 48 h). Univariable and multivariable logistic regression analyses were conducted to identify predictors of postoperative hyperglycemia. Results: Hyperglycemia occurred in 65.9% of all patients. Univariable analysis identified fasting plasma glucose (FPG), mean postoperative glucose, number of antidiabetic medications, and glucose variability as significant predictors (*p* < 0.05). In multivariable analysis adjusted for HbA1c, glycoalbumin, and glucose variability, FPG [odds ratio (OR): 1.07; 95% confidence interval (CI): 1.01–1.14; *p* = 0.024], mean glucose (OR: 1.12; 95% CI: 1.02–1.23; *p* = 0.017), and glucose variability (OR: 1.19; 95% CI: 1.05–1.35; *p* = 0.008) remained independently associated with hyperglycemia. Conclusions: CGM revealed a high incidence of early postoperative hyperglycemia despite conventional sliding-scale insulin therapy. These findings highlight the limitations of current glycemic protocols and underscore the potential of CGM as a diagnostic tool to guide individualized glucose management. Future studies should evaluate whether CGM-guided interventions can improve surgical outcomes, particularly in reducing SSI risk among high-risk diabetic patients.

## 1. Introduction

Surgical site infections (SSIs), including periprosthetic joint infections (PJIs), are serious and potentially intractable complications following total hip and knee arthroplasty (THA and TKA). Once established, these infections place a substantial burden on patients, healthcare providers, and the healthcare system. Risk factors for PJI are broadly categorized into intrinsic and extrinsic types. Intrinsic factors include patient-related conditions such as diabetes mellitus, rheumatoid arthritis, human immunodeficiency virus infection (including hemophilia), chronic hemodialysis, advanced age, and malnutrition, while extrinsic factors are associated with surgical and procedural variables [1].

Among intrinsic factors, diabetes mellitus is a well-established contributor to increased susceptibility to SSIs in the context of arthroplasty [2,3]. Current guidelines emphasize the importance of preoperative glycemic optimization, recommending that glycated hemoglobin A1c (HbA1c) be maintained below 7.5–8.0% to reduce infection risk [4]. However, perioperative glycemic management involves more than preoperative control alone. Notably, postoperative blood glucose levels ≥ 200 mg/dL on the first postoperative day have been associated with a twofold increase in SSI risk [5], underscoring the importance of glycemic control during the early postoperative period [6]. In addition, increased glucose variability has been linked to impaired wound healing and immune function, potentially elevating infection risk [7].

Despite these insights, few studies have used continuous glucose monitoring (CGM) to assess postoperative glycemic profiles in arthroplasty patients [8,9]. In most clinical settings, perioperative glucose management is based on intermittent blood glucose checks and reactive sliding-scale insulin protocols. These approaches may inadequately reflect actual glycemic dynamics and may fail to identify transient but clinically relevant hyperglycemia, even under the supervision of diabetologists. While some reports have described CGM in surgical populations, to our knowledge, no study has specifically evaluated predictors of postoperative hyperglycemia using CGM-derived parameters in patients with diabetes undergoing arthroplasty.

Therefore, the objective of this study was to characterize early postoperative glycemic patterns using CGM in patients with type 2 diabetes mellitus undergoing THA or TKA and to identify clinical and perioperative factors associated with postoperative hyperglycemia. By revealing the limitations of current glycemic management protocols, particularly the sliding-scale approach, this study aims to clarify the potential role of CGM as a tool for evaluating and ultimately improving perioperative glucose management strategies.

## 2. Materials and Methods

### 2.1. Participants

This retrospective observational study was approved in advance by the Institutional Review Board (IRB) of our institution. Written informed consent was obtained from all participants prior to their inclusion in the study and the publication of the results.

Between January 2021 and September 2022, a total of 360 patients underwent lower extremity arthroplasty at our institution, including 174 primary and 12 revision THAs and 168 primary and 6 revision TKAs. Of these, patients with a preoperative diagnosis of type 2 diabetes mellitus who were receiving management by a diabetologist were eligible for inclusion. Patients were excluded if they did not undergo postoperative CGM or if their CGM data were incomplete or unavailable. As a result, 41 patients with type 2 diabetes mellitus who underwent lower extremity arthroplasty (THA: 11 primary and 4 revisions; TKA: 25 primary and 1 revision) were included in the final analysis.

To minimize dietary effects on glucose levels, all patients were in structed to refrain from eating after 21:00 and from drinking water after midnight on the day prior to surgery. Oral intake was resumed at 8:00 on postoperative day 1. In accordance with Japanese perioperative guidelines, oral hypoglycemic agents, including biguanides and sodium-glucose cotransporter 2 (SGLT2) inhibitors, were discontinued 2 and 3 days before surgery, respectively. Other antidiabetic agents were withheld on the day of surgery.

### 2.2. Measurement of Blood Glucose Levels

Perioperative glycemic control was supervised by a diabetologist using a sliding-scale insulin protocol (Table 1). Blood glucose levels were routinely measured using the Medisafe FIT^®^ Blood Glucose Meter (Terumo Corporation, Tokyo, Japan) at 6:00, upon returning to the ward from the operating room, 18:00, and 23:00 on the day of surgery, and, subsequently, at 8:00 (before breakfast), 12:00 (before lunch), 18:00 (before dinner), and 21:00 (at bedtime) on postoperative day 1.

Additionally, postoperative interstitial glucose levels were continuously monitored using the FreeStyle Libre Pro^®^ system (Abbott Diabetes Care Inc., Alameda, CA, USA). This CGM system utilizes a subcutaneous sensor applied to the upper arm and has been validated as a reliable surrogate for blood glucose measurements [10]. The device records glucose levels every 15 min and is capable of continuous monitoring for up to 14 days.

CGM monitoring commenced immediately after patients returned to the ward following surgery and continued for 48 h postoperatively. All measurements were obtained under inpatient management and CGM data were retrospectively extracted for analysis. To ensure data accuracy, only readings from 12 to 48 h after sensor application were included in the analysis, as earlier data are known to be potentially unstable [11].

Patients were retrospectively classified into two groups according to postoperative glycemic status measured by CGM: the normoglycemia group, in which all glucose levels remained <200 mg/dL during the 48-h monitoring period, and the hyperglycemia group, in which glucose levels reached or exceeded ≥200 mg/dL at least once during the same period.

The proportion of time during which glucose levels were ≥200 mg/dL was calculated using the following formula:

$$Percentage=\;\frac{minutes\geq 200\;mg/dL}{36\;h\times 60\;min}\times 100$$

### 2.3. Statistical Analysis

All continuous variables were tested for normality using the D’Agostino–Pearson omnibus test (GraphPad Prism version 8; GraphPad Software, Inc., San Diego, CA, USA). Variables with a normal distribution are expressed as the mean ± standard deviation (SD), whereas non-normally distributed variables are expressed as the median with interquartile range (IQR).

To identify independent predictors of postoperative hyperglycemia, univariable and multivariable logistic regression analyses were performed. Odds ratios (ORs) with 95% confidence intervals (CIs) were calculated. Variables entered into the multivariable models were selected based on clinical relevance and to avoid multicollinearity. Each model was adjusted for HbA1c, glycoalbumin (GA), and postoperative blood glucose variability [12,13,14].

The goodness-of-fit and model calibration were confirmed using standard diagnostic statistics, including the Nagelkerke R^2^ and Hosmer–Lemeshow tests, which indicated adequate model performance. All logistic regression analyses were conducted using IBM SPSS Statistics, version 29.0.1.0 (IBM Corp., Armonk, NY, USA).

## 3. Results

In total, 41 patients, comprising 11 men and 30 women, were included in this study. No cases of SSI or PJI were observed during the study period. Sociodemographic and clinical characteristics are summarized in Table 2. The mean (±SD) age at the time of surgery was 73.9 ± 9.6 years, the mean duration of diabetes was 8.5 ± 8.5 years, and the mean preoperative body mass index (BMI) was 26.2 ± 4.2 kg/m^2^. The mean preoperative HbA1c was 6.5 ± 0.5%.

With respect to underlying joint pathology, 10 patients had osteoarthritis of the hip, 1 had osteonecrosis of the femoral head, 24 had knee osteoarthritis, 1 had rheumatoid arthritis of the knee, 4 underwent revision THA for non-infectious causes, and 1 underwent revision TKA for a non-infectious cause. Most patients were managed with oral antidiabetic agents, while insulin therapy was administered in two cases.

**Figure 1:**
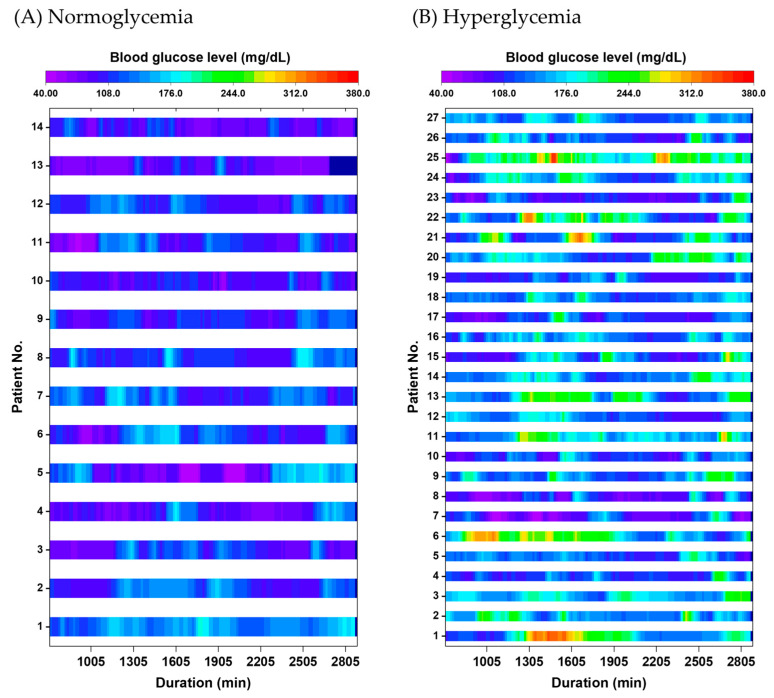
Split heatmaps showing 48 h postoperative blood glucose levels measured using continuous glucose monitoring (CGM) in (**A**) normoglycemia and (**B**) hyperglycemia groups. Each row represents an individual patient, with time (hours) on the horizontal axis and glucose levels displayed using a continuous color scale. Periods with glucose ≥ 200 mg/dL are visualized primarily in yellow-green to red, corresponding to moderate to marked hyperglycemia. Overall, 65.9% of patients experienced at least one episode of glucose ≥ 200 mg/dL during the monitoring period.

CGM monitoring during the 48 h postoperative period revealed a mean blood glucose level of 135.4 ± 4.2 mg/dL and a median (IQR) glucose variability of 32.1 (26.3–41.9) mg/dL. Overall, 27 patients (65.9%) experienced at least one episode of blood glucose ≥ 200 mg/dL within the 48 h period (Figure 1). Regarding the proportion of time spent in a hyperglycemic state, 14 patients (34.1%) had no glucose readings ≥ 200 mg/dL, 14 (34.1%) exhibited hyperglycemia for less than 10% of the monitoring period, 5 (12.2%) for 10–20% (equivalent to 4.8–9.6 h), and 8 (19.5%) for more than 20% (>9.6 h). The maximum observed proportion was 60.4% (21.7 h), indicating that glycemic control remained suboptimal in some patients despite the application of a sliding-scale insulin protocol.

In univariable analyses comparing the normoglycemia and hyperglycemia groups, significant differences were observed in the following variables (Table 3): the number of preoperative antidiabetic medications (OR, 2.03; 95% CI, 1.06–3.88; *p* = 0.033), mean postoperative blood glucose level (OR, 1.09; 95% CI, 1.03–1.14; *p* = 0.003), and postoperative blood glucose variability (OR, 1.16; 95% CI, 1.04–1.30; *p* = 0.007).

In the multivariable logistic regression model adjusted for HbA1c, GA, and glucose variability, the following factors remained independently associated with postoperative hyperglycemia: FPG (OR, 1.07; 95% CI, 1.01–1.14; *p* = 0.024), mean postoperative blood glucose level (OR, 1.12; 95% CI, 1.02–1.23; *p* = 0.017), and postoperative glucose variability (OR, 1.19; 95% CI, 1.05–1.35; *p* = 0.008) (Table 3).

## 4. Discussion

Diabetes mellitus is a well-established host-related risk factor for SSIs and PJIs, primarily due to its adverse effects on immune function. Elevated perioperative blood glucose levels can impair neutrophil activity and reduce antibody production, thereby increasing the risk of postoperative infection in joint arthroplasty patients [1,2,3,4,5,6,7,15,16,17,18,19].

In lower extremity arthroplasty, the prevalence of diabetes has been reported at 20.6%, with 40.9% of cases previously undiagnosed [18]. When including prediabetes, 58.9% of patients were found to have abnormal glycemic status, supporting recommendations for universal preoperative blood glucose screening in elective arthroplasty candidates. Moreover, nondiabetic stress-induced hyperglycemia has been observed in more than 50% of patients undergoing arthroplasty [19], underscoring the importance of perioperative glycemic control in all surgical patients, regardless of known diabetic status.

Glycemic control during the first 48 h after surgery is particularly critical [6], as postoperative blood glucose levels ≥ 200 mg/dL on the first postoperative day have been associated with a twofold increase in SSI risk [5]. Additionally, greater blood glucose variability has been shown to further elevate the risk of infection [7]. However, few studies have evaluated the use of CGM in the postoperative setting [8,9], highlighting the need to assess its clinical utility [16].

In Japan, perioperative glycemic control is typically managed through intermittent blood glucose measurements three to four times daily and the use of sliding scale insulin protocols, as shown in Table 1. In the present study, 65.9% of patients experienced blood glucose levels exceeding 200 mg/dL within 48 h postoperatively, despite a preoperative diagnosis of type 2 diabetes mellitus and management by diabetologists (Figure 1). This suggests that sliding scale insulin protocols may be insufficient to manage dynamic glycemic fluctuations during the early postoperative period.

Several previous studies have proposed HbA1c as a predictor of SSIs and PJIs. For example, Stryker et al. [20] reported that HbA1c ≥ 6.7% was associated with an increased risk of wound complications after primary arthroplasty, showing an OR of 9.0 (95% CI, 1.14–71.20; *p* = 0.03). Similarly, Hwang et al. [21] found an association between HbA1c ≥ 8% and superficial SSIs following TKA, reporting an OR of 6.1 (95% CI, 1.6–23.4; *p* = 0.008). The second International Consensus Meeting (ICM) on Musculoskeletal Infection also recommended an HbA1c threshold of 7.5 to 8% as a predictor of SSI and PJI risk [4].

However, in the present study, HbA1c was not a significant predictor of post operative hyperglycemia in the multivariable analysis (Table 3). This finding is consistent with a systematic review and meta-analysis by Shohat et al. [22], which found no consensus on a definitive HbA1c cutoff for SSI risk. A study conducted by Hagedorn et al. [23] also failed to demonstrate a consistent association between HbA1c and infection outcomes, emphasizing that perioperative glycemic control may be more important than preoperative HbA1c levels alone.

Our data indicate that high preoperative FPG, elevated postoperative mean glucose, and increased glucose variability are more relevant predictors of early postoperative hyperglycemia than HbA1c alone. High FPG may reflect underlying insulin resistance or beta cell dysfunction, which impairs the patient’s metabolic flexibility and ability to counteract the hyperglycemic effects of surgical stress. These findings align with previous evidence linking preoperative FPG to increased SSI risk after arthroplasty [24]. In our cohort, patients were preoperatively well controlled in terms of HbA1c, but those with high FPG may have had insufficient suppression of nocturnal hepatic glucose output. This impaired regulation, particularly during perioperative hormonal surges such as cortisol and catecholamines, likely contributed to both elevated mean glucose and the persistence of postoperative hyperglycemia.

Glycemic variability has, in turn, been associated with oxidative stress, endothelial dysfunction, and impaired immune function [25]. These mechanisms may directly compromise wound healing and immune surveillance, leading to increased susceptibility to postoperative complications. Moreover, reactive sliding scale insulin therapy may inadvertently exacerbate glucose fluctuations in patients with diminished stress adaptability.

In this study, patients were required to fast from the night before surgery until the evening of the operative day and resumed oral intake at breakfast the following morning. Although sliding-scale insulin was initiated at the time of refeeding due to concerns over fluctuating food intake, our data showed that 54% of patients consumed more than half of their first postoperative meal, suggesting relatively stable intake. Given the generally good condition of arthroplasty patients, this finding supports the feasibility of earlier transition to intensive insulin therapy [26], combining basal and premeal short acting insulin, rather than continued reliance on sliding scale regimens. Such a strategy may help reduce the duration and severity of postoperative hyperglycemia, particularly in the first 48 h, and it underscores the importance of early collaboration with diabetes specialists.

Taken together, elevated FPG, increased mean glucose, and high glycemic variability may not only reflect inadequate glycemic control but also play a mechanistic role in the development of postoperative complications. Although our study did not include any observed cases of SSI or PJI, previous reports linking hyperglycemia and glucose variability with infection outcomes suggest that these factors may be clinically relevant [5,7]. Identifying high-risk patients based on dynamic glycemic parameters and introducing individualized, CGM-guided insulin strategies may therefore be essential for improving perioperative outcomes.

This study has several limitations. The sample size was relatively small, which may have limited the statistical power of the multivariable analyses and the generalizability of the findings. However, the use of CGM provided high-resolution insight into perioperative glucose dynamics that cannot be captured by intermittent measurements alone.

Potential sources of bias should also be acknowledged. Because this was a retrospective observational study including only patients with a known diagnosis of type 2 diabetes mellitus, selection bias cannot be excluded. In addition, despite excluding the initial 12 h of CGM data to ensure sensor stabilization, measurement bias related to the inherent characteristics of the device may still exist. Moreover, due to the limited sample size, residual confounding from unmeasured variables could not be completely eliminated.

Our cohort included both hip and knee arthroplasty cases, encompassing both primary and revision procedures. Although these differences may contribute to variability in surgical stress and postoperative glycemic profiles [27], Jämsen et al. [28] previously reported that glycemic responses were comparable following hip and knee arthroplasty. Nevertheless, the limited sample size in our study precluded a reliable subgroup analysis by joint type or procedure category. Future studies with larger and more homogeneous cohorts are warranted to clarify these associations.

Transient low-glucose readings were observed in a subset of patients on CGM; however, no clinical symptoms of hypoglycemia were noted in any case. It should be noted that the FreeStyle Libre Pro^®^ system has been reported to slightly underestimate glucose levels in the lower range compared with capillary blood glucose measurements [29]. Therefore, these apparent hypoglycemic episodes likely reflect the inherent characteristics of the sensor rather than true biochemical hypoglycemia. To further ensure data accuracy, only readings obtained 12–48 h after sensor application were included in the analysis, during which the device provides stable measurements [11].

Additionally, only patients with type 2 diabetes were included; undiagnosed diabetes and stress hyperglycemia in non-diabetic patients were not assessed. Future studies should include broader populations and evaluate glycemic management strategies in those without a formal diabetes diagnosis. Furthermore, our institutional protocol involves relatively prolonged pre- and postoperative fasting periods, which may not align with current recommendations such as those from enhanced recovery after surgery (ERAS) guidelines [30]. Extended fasting may influence perioperative glycemic variability and nutritional status, both of which are important factors in surgical recovery and infection prevention. Although this protocol was applied uniformly across all patients in this study, future investigations should examine the potential impact of early nutritional interventions on glycemic control and clinical outcomes. Finally, the absence of SSI or PJI events in this study precludes conclusions regarding the direct impact of glycemic control on infection incidence.

Despite these limitations, this study provides compelling evidence that CGM reveals a high rate of early postoperative hyperglycemia in patients with type 2 diabetes undergoing arthroplasty. These findings highlight the limitations of conventional sliding scale insulin protocols and support the integration of CGM-based approaches to optimize perioperative glycemic control. Although infection outcomes were not evaluated directly, the observed glycemic patterns and their associations with known risk factors suggest that tighter glucose management during the early postoperative period may play a meaningful role in preventing complications. Implementing individualized glycemic strategies informed by CGM could therefore contribute not only to improved metabolic outcomes but also to safer surgical care.

## 5. Conclusions

CGM revealed a high incidence of early postoperative hyperglycemia despite conventional sliding-scale insulin therapy. These findings highlight the limitations of current glycemic protocols and underscore the potential of CGM as a diagnostic tool to guide individualized glucose management. Future studies should evaluate whether CGM-guided interventions can improve surgical outcomes, particularly in reducing SSI risk among high-risk diabetic patients.

## Figures and Tables

**Table 1:** Sliding-scale insulin protocol for postoperative blood glucose management.

Blood Glucose Level (mg/dL)	Human Insulin (Humulin^®^ R) Dose (Unit)
150–199	2
200–249	4
250–299	6
300–349	8
≥350	10

**Table 2:** Baseline clinical demographics of patients classified into normoglycemia and hyperglycemia groups based on postoperative glycemic status measured by continuous glucose monitoring (CGM).

	Overall (*n* = 41)	Normoglycemia (*n* = 14)	Hyperglycemia (*n* = 27)
Age (years) *	73.9 ± 9.6	71.2 ± 10.5	75.3 ± 9.0
BMI (kg/m^2^) *	26.2 ± 4.2	25.7 ± 5.1	26.4 ± 3.8
Disease duration (years) ^†^	5.0 (2.3–13.0)	4.0 (1.0–14.5)	7.0 (3.0–12.0)
HbA1c (%) *	6.5 ± 0.5	6.4 ± 0.5	6.6 ± 0.5
GA (%) ^†^	15.2 (14.1–17.9)	14.4 (13.6–16.3)	15.3 (14.8–18.6)
FPG (mg/dL) ^†^	103.0 (94.0–119.0)	101.5 (90.3–106.0)	111.0 (96.0–130.0)
CPR (ng/mL) ^†^	2.4 (1.6–2.9)	2.5 (1.6–3.7)	2.2 (1.5–2.7)
CPI ^†^	2.0 (1.4–3.0)	2.5 (1.9–3.5)	1.9 (1.2–2.6)
UCPR (µg/day) ^†^	52.9 (35.3–86.2)	52.6 (38.4–89.9)	53.4 (29.3–85.4)
eGFR (mL/min) *	70.0 ± 20.0	65.2 ± 21.7	72.6 ± 19.0
Ualb (µg/min) ^†^	18.2 (7.3–60.5)	17.2 (4.9–122.4)	19.1 (7.5–53.6)
Operation time (min) ^†^	81.0 (69.0–91.0)	80.0 (64.3–88.8)	85.0 (71.0–92.0)
Postoperative mean blood glucose level (mg/dL) *	135.4 ± 31.3	109.8 ± 15.1	148.6 ± 29.3
Postoperative blood glucose variability (mg/dL) ^†^	32.1 (26.3–41.9)	23.7 (18.2–29.3)	35.9 (30.4–47.1)
Number of diabetic drugs *	1.8 ± 1.1	1.2 ± 1.0	2.0 ± 1.1
Type of diabetic drugs	Number of users		
Non-Medication	7	4	3
Biguanides	17 (23.6%)	3	14
Sulfonylureas	1 (1.4%)	1	0
Rapid-acting insulin secretagogues	2 (2.8%)	0	2
Alpha-glucosidase inhibitors	4 (5.6%)	1	3
SGLT2 inhibitors	15 (20.8%)	4	11
DPP-4 inhibitors	26 (36.1%)	6	20
GLP-1 receptor agonist	5 (6.9%)	2	3
Insulin	2 (2.8%)	0	2

BMI: body mass index, HbA1c: hemoglobin A1c, GA: glycoalbumin, FPG: fasting plasma glucose, CPR: C-peptide, CPI: C-peptide index, UCPR: urine C-peptide, eGFR: estimated glomerular filtration rate, Ualb: urine albumin, * The values are presented as mean ± standard deviation, ^†^ The values are presented as median (interquartile range).

**Table 3:** Odds ratio estimates for blood glucose levels ≥ 200 mg/dL within 48 h postoperatively, derived from univariable and multivariable logistic regression models.

	Unadjusted		Multivariable-Adjusted ^a^	
	OR (95% CI)	*p*	OR (95% CI)	*p*
Age	1.05 (0.98–1.12)	0.202	1.02 (0.93–1.12)	0.609
Gender	1.14 (0.27–4.84)	0.856	1.41 (0.21–9.64)	0.726
BMI	1.04 (0.89–1.22)	0.609	1.22 (0.94–1.60)	0.140
Disease duration	1.03 (0.94–1.12)	0.563	0.94 (0.82–1.09)	0.420
HbA1c	2.84 (0.68–11.82)	0.152	5.46 (0.718–41.47)	0.101
GA	1.24 (0.92–1.67)	0.167	1.01 (0.69–1.50)	0.951
FPG	1.03 (1.00–1.07)	0.094	1.07 (1.01–1.14)	0.024 *
CPR	0.84 (0.62–1.13)	0.251	1.06 (0.79–1.42)	0.712
CPI	0.72 (0.41–1.25)	0.240	0.96 (0.68–1.36)	0.820
UCPR	1.00 (0.99–1.02)	0.601	1.00 (0.97–1.03)	0.985
eGFR	1.02 (0.99–1.06)	0.266	1.01 (0.96–1.06)	0.858
Ualb	1.00 (0.99–1.01)	0.447	1.00 (0.98–1.01)	0.509
Mean blood glucose level	1.09 (1.03–1.14)	0.003 *	1.12 (1.02–1.23)	0.017 *
Operation time	1.01 (0.97–1.06)	0.500	1.04 (0.97–1.11)	0.290
Number of diabetic drugs	2.03 (1.06–3.88)	0.033 *	1.61 (0.72–3.61)	0.245
Blood glucose variability	1.16 (1.04–1.30)	0.007 *	1.19 (1.05–1.35)	0.008 *

BMI: body mass index, GA: glycoalbumin, FPG: fasting plasma glucose, CPR: C-peptide, CPI: C-pep tide index, UCPR: urine C-peptide, eGFR: estimated glomerular filtration rate, Ualb: urine albumin, *: *p* < 0.05. ^a^ Adjusted for HbA1c, GA, and blood glucose variability.

## Data Availability

All data analyzed during this study are included in this article.

## References

[1] The Japanese Orthopaedic Association (2015). Japanese Orthopaedic Association Clinical Practice Guideline on the Prevention of Postoperative Infection of Osteoarthrotomy.

[2] Marchant M.H., Viens N.A., Cook C., Vail T.P., Bolognesi M.P. (2009). The impact of glycemic control and diabetes mellitus on perioperative outcomes after total joint arthroplasty. J. Bone Jt. Surg..

[3] Kunutsor S.K., Whitehouse M.R., Blom A.W., Beswick A.D., INFORM Team (2016). Patient-related risk factors for periprosthetic joint infection after total joint arthroplasty: A systematic review and meta-analysis. PLoS ONE.

[4] ICM The International Consensus Meeting on Infection (ICMI). Proceedings of the Second International Consensus Meeting on Musculoskeletal Infection.

[5] Mraovic B., Suh D., Jacovides C., Parvizi J. (2011). Perioperative hyperglycemia and postoperative infection after lower limb arthroplasty. J. Diabetes Sci. Technol..

[6] Furnary A.P., Wu Y. (2006). Eliminating the diabetic disadvantage: The Portland Diabetic Project. Semin. Thorac. Cardiovasc. Surg..

[7] Goh G.S., Shohat N., Abdelaal M.S., Small I., Thomas T., Ciesielka K.A., Parvizi J. (2022). Serum glucose variability increases the risk of complications following aseptic revision hip and knee arthroplasty. J. Bone Jt. Surg. Am..

[8] Maeda Y., Nakamura N., Tsujimoto T., Sugano N. (2019). Higher blood glucose and larger fluctuations detected postoperatively using continuous glucose monitoring: A preliminary study following total knee or hip arthroplasty. J. Exp. Orthop..

[9] Mraovic B., Simurina T., Joseph J.I. (2021). Perioperative hyperglycemia in elective arthroplasties. Should we do better?. Acta Anaesthesiol. Scand..

[10] Rebrin K., Sheppard N.F., Steil G.M. (2010). Use of subcutaneous interstitial fluid glucose to estimate blood glucose: Revisiting delay and sensor offset. J. Diabetes Sci. Technol..

[11] Alva S., Bailey T., Brazg R., Budiman E.S., Castorino K., Christiansen M.P., Forlenza G., Kipnes M., Liljenquist D.R., Liu H. (2022). Accuracy of a 14-day factory-calibrated continuous glucose monitoring system with advanced algorithm in pediatric and adult population with diabetes. J. Diabetes Sci. Technol..

[12] Sartore G., Chilelli N.C., Burlina S., Di Stefano P., Piarulli F., Fedele D., Mosca A., Lapolla A. (2012). The importance of HbA1c and glucose variability in patients with type 1 and type 2 diabetes: Outcome of continuous glucose monitoring (CGM). Acta Diabetol..

[13] Škrha J., Šoupal J., Škrha J., Prázný M. (2016). Glucose variability, HbA1c and microvascular complications. Rev. Endocr. Metab. Disord..

[14] Sonoda S., Okada Y., Torimoto K., Sugai K., Uemura F., Tanaka K., Hajime M., Mori H., Tanaka Y. (2020). Correlations between glycemic parameters obtained from continuous glucose monitoring and hemoglobin A1c and glycoalbumin levels in type 2 diabetes mellitus. J. UOEH.

[15] Olsen M.A., Nepple J.J., Riew K.D., Lenke L.G., Bridwell K.H., Mayfield J., Fraser V.J. (2008). Risk factors for surgical site infection following orthopaedic spinal operations. J. Bone Jt. Surg. Am..

[16] Lamanna D.L., McDonnell M.E., Chen A.F., Gallagher J.M. (2022). Perioperative identification and management of hyperglycemia in orthopaedic surgery. J. Bone Jt. Surg. Am..

[17] Akiboye F., Rayman G. (2017). Management of hyperglycemia and diabetes in orthopedic surgery. Curr. Diabetes Rep..

[18] Shohat N., Goswami K., Tarabichi M., Sterbis E., Tan T.L., Parvizi J. (2018). All patients should be screened for diabetes before total joint arthroplasty. J. Arthroplast..

[19] Rudy M.D., Ahuja N.K., Aaronson A.J. (2018). Diabetes and hyperglycemia in lower-extremity total joint arthroplasty: Clinical epidemiology, outcomes, and management. JBJS Rev..

[20] Stryker L.S., Abdel M.P., Morrey M.E., Morrow M.M., Kor D.J., Morrey B.F. (2013). Elevated postoperative blood glucose and preoperative hemoglobin A1C are associated with increased wound complications following total joint arthroplasty. J. Bone Jt. Surg. Am..

[21] Hwang J.S., Kim S.J., Bamne A.B., Na Y.G., Kim T.K. (2015). Do glycemic markers predict occurrence of complications after total knee arthroplasty in patients with diabetes?. Clin. Orthop. Relat. Res..

[22] Shohat N., Muhsen K., Gilat R., Rondon A.J., Chen A.F., Parvizi J. (2018). Inadequate glycemic control is associated with increased surgical site infection in total joint arthroplasty: A systematic review and meta-analysis. J. Arthroplast..

[23] Hagedorn J.M., Bendel M.A., Hoelzer B.C., Aiyer R., Caraway D. (2023). Preoperative hemoglobin A1c and perioperative blood glucose in patients with diabetes mellitus undergoing spinal cord stimulation surgery: A literature review of surgical site infection risk. Pain Pract..

[24] Kieruzel N., Sethi S., Nair V., Wolf J.M., Strelzow J.A. (2024). Do preoperative glucose levels predict risk of complications in orthopaedic surgery?. Eur. J. Orthop. Surg. Traumatol..

[25] Wang X., Cao Y. (2025). A Narrative review: Relationship between glycemic variability and emerging complications of diabetes mellitus. Biomolecules.

[26] Kramer C.K., Zinman B., Retnakaran R. (2013). Short-term intensive insulin therapy in type 2 diabetes mellitus: A systematic review and meta-analysis. Lancet Diabetes Endocrinol..

[27] Sonohata M., Tsunoda K., Kugisaki H., Someya S., Honke H., Komine M., Kitajima M., Mawatari M., Hotokebuchi T. (2009). Surgical stress differences between total hip arthroplasty and total knee arthroplasty. Int. J. Med. Med. Sci..

[28] Jämsen E., Nevalainen P.I., Eskelinen A., Kalliovalkama J., Moilanen T. (2015). Risk factors for perioperative hyperglycemia in primary hip and knee replacements. Acta Orthop..

[29] Fokkert M.J., van Dijk P.R., Edens M.A., Abbes S., de Jong D., Slingerland R.J., Bilo H.J. (2017). Performance of the FreeStyle Libre Flash glucose monitoring system in patients with type 1 and 2 diabetes mellitus. BMJ Open Diabetes Res. Care.

[30] Melnyk M., Casey R.G., Black P., Koupparis A.J. (2011). Enhanced recovery after surgery (ERAS) protocols: Time to change practice?. Can. Urol. Assoc. J..

